# 3D Flow in the Venom Channel of a Spitting Cobra: Do the Ridges in the Fangs Act as Fluid Guide Vanes?

**DOI:** 10.1371/journal.pone.0061548

**Published:** 2013-05-06

**Authors:** Michael Triep, David Hess, Humberto Chaves, Christoph Brücker, Alexander Balmert, Guido Westhoff, Horst Bleckmann

**Affiliations:** 1 Institut für Mechanik und Fluiddynamik, TU Bergakademie Freiberg, Freiberg, Germany; 2 Institut für Zoologie, Universität Bonn, Bonn, Germany; University of California, Irvine, United States of America

## Abstract

The spitting cobra *Naja pallida* can eject its venom towards an offender from a distance of up to two meters. The aim of this study was to understand the mechanisms responsible for the relatively large distance covered by the venom jet although the venom channel is only of micro-scale. Therefore, we analysed factors that influence secondary flow and pressure drop in the venom channel, which include the physical-chemical properties of venom liquid and the morphology of the venom channel. The cobra venom showed shear-reducing properties and the venom channel had paired ridges that span from the last third of the channel to its distal end, terminating laterally and in close proximity to the discharge orifice. To analyze the functional significance of these ridges we generated a numerical and an experimental model of the venom channel. Computational fluid dynamics (CFD) and Particle-Image Velocimetry (PIV) revealed that the paired interior ridges shape the flow structure upstream of the sharp 90° bend at the distal end. The occurrence of secondary flow structures resembling Dean-type vortical structures in the venom channel can be observed, which induce additional pressure loss. Comparing a venom channel featuring ridges with an identical channel featuring no ridges, one can observe a reduction of pressure loss of about 30%. Therefore it is concluded that the function of the ridges is similar to guide vanes used by engineers to reduce pressure loss in curved flow channels.

## Introduction

Spitting cobras belong to the Elapidae, a large family of venomous snakes that includes mambas, taipans, and death adders (e.g., [1]). Several species of African and Asian spitting cobras of the genera *Naja* and *Hemachatus* expel their venom as a fast, pulsed stream that leaves the fangs at a nearly right angle [2], [3]. The spitting behaviour of cobras evolved independently among different cobra species [4], [5]. Venom spitting is used as a defensive strategy against vertebrates [2], [3], [6]. The venom stream is aimed at the face of an offender, where the venom causes severe pain if it hits the eyes [7], [8], [9]. Spitting cobras not only aim at a target but in addition adjust their venom distribution to target distance by rapid head movements [10]. The venom delivery system of spitting cobras possesses several morphological adaptations, distinguishing them from non-spitting cobra species. The discharge orifice for example has a more circular shape, enabling them to expel the venom forward rather than downward [2], [6]. Hence, the venom travels through a sharp 90° bend before leaving the fang. Furthermore, the venom channel of the fang has ridges that are unique to spitting cobras. These usually paired ridges span from the last third of the total length of the venom channel to its distal end, and end just before the sharp 90° bend that redirects the flow towards the discharge orifice [11]. Most likely, the ridges are an adaptation to the requirements of venom spitting because they are not found in non-spitting cobras [9]. Other biological systems where a high-speed jet occurs can be found in the archerfish and the snapping shrimp. The archerfish can shape the tongue in a way that a channel is formed. In a similar manner the claws of a snapping shrimp have a tapered V-shaped structure. Two technical applications of high-speed jets with relevance to our investigations are the medical-surgical water knife and fuel injection systems with a sharp bend. The aim of this study was to investigate the function of the ridges in the venom channel of the spitting cobra *Naja pallida*, to determine how the venom jet is generated and what level of pressure loss must be compensated for by the venom gland contraction to achieve high jet velocities at the exit. Our focus lied on the establishment of secondary flow in the channel, specifically with respect to secondary flow in curved passages (Dean vortices, [12]) and its influence on pressure loss. Next, the fluid-dynamic model of the venom channel is described. Thereafter, the numerical and experimental methods of the present study are delineated. Finally, the results are presented and discussed.

## Materials and Methods

### Investigation of the venom properties and morphology of the venom channel

Adult spitting cobras (*Naja pallida, N = 7*) were kept in glass containers at a temperature of 20–27°C and a humidity of at least 70%. *Naja pallida* were captive bred. All snakes were regularly fed with small to medium sized live rodents and given water ad libitum. An authorization to house the cobras has been obtained. All animals were housed at the Institute of Zoology of the University of Bonn in accordance with regional laws to the keeping of venomous snakes as well as applying rules for laboratory animals. We did not have IACUC approval of experiments which is neither required nor a common procedure for experiments in Europe. All experiments were according to the Principles of Animal Care. Animals were not anaesthetized. Snakes were gently held behind the neck before a jar covered with a para-film was presented in front of their mouth. The snakes readily bit through the para-film injected their venom into the jar. After milking, the venom is immediately transferred into small plastic vessels (Eppendorf GmbH, Germany). The volume of venom yielded in each milking process is measured. Plastic vessels are stored at 10°C to prevent any decay of the venom until measurement. Venomous snakes substitute their fangs every 6–8 weeks. Thus, the snakes' mouths were inspected every 4–6 weeks in order to pick the substituted fang. The substituted fang normally gets lost out of the fang membranes with the snakes next meal. We picked the loose fang out of the membranes with a forceps, cleaned in an ultrasonic cleaner and air-dried for the morphological investigations. For each individual, the viscosity of the venom is measured in a rotational rheometer (Bohlin Gemini 2, Bohlin Ltd., USA). The venom is transferred directly into the specimen chamber of the rheometer. The specimen chamber is immediately closed and sealed with a special solvent trap to prevent dehydration of the venom. For each measurement, a volume of about 150 µl venom liquid is used. Measurements were conducted at a temperature of 20°C and at continuously increasing shear. Measurements lasted for about 15 minutes. They were repeated three times (with breaks of five minutes in between two measurements) to verify that the values were reproducible.

The surface tension of the venom was measured in a tensiometer (OCA 30, DataPhysics Instruments GmbH, Filderstadt, Germany) at 20°C. About 100 µl of venom was filled into a syringe connected to a cannula (radius 1 mm). Small droplets of venom were pumped out of the cannula to create a hanging droplet. The droplet was photographed with the OCA camera and its surface tension was calculated with the OCA software. Fifteen measurements were made for each of two individuals. In addition, the venom was weighed with a high precision scale (Bp 110 S, Sartorius AG, Germany). Thereafter, the density ρ of the venom was calculated according to the equation ρ = G/V, with G = weight and V = volume. All measurements were conducted at 20°C room temperature and normal air pressure. Density was expressed in g cm^−3^. Ten measurements were made for each individual (N = 2).

The fangs of the spitting cobra were placed in a small plastic vessel and stabilized with cotton watting. The fangs were scanned from tip to base with a micro-computer tomograph (MCT, μCT 20, Scanco, Bassersdorf, Germany) and visualized in three-dimensions with the software Amira® (Amirasoft ltd., Germany). Also, scanning electron microscopy (SEM) was used as a control for the resolution of the MCT data. For scanning electron microscopy, we prepared a parasagittal section of dried fangs by using a diamond drill to expose the venom channel. The fangs were placed on aluminum stabs using a liquid conductant graphite. Afterwards fangs were sputter-coated with silver and scanned in a Cambridge Stereoscan Microscope (Cambridge Instruments, Oxford, England).

### The fluid-dynamical model of the venom channel

Venom-gland contraction provides the only force for venom expulsion [13], [14]. Young et al. [15] measured the venom pressure at the fang tips of a spitting cobra (*N. pallida*). While this has been done successfully in the past, it is impossible to measure in vivo the pressure build-up at the entrance of the venom channel. This information – which is important for the analysis of the flow in the fang model – can, however, be obtained with a numerical simulation, provided that the geometry of the venom channel and the flow rates of the venom are known. Therefore, the real venom channel was transferred into a model with the aid of computer micro-tomography. The 3D structure of the channel in the model was first reconstructed from the images of the cross-sections of a cobra fang and then smoothed mathematically. Due to its complex geometry the channel was subdivided along its long axis into three parts (cf. results section). The 3D computer-aided design model of the venom channel was created and transferred into a computational grid using a grid generation tool (ANSYS 12.1 ICEM CFD, see Appendix S1). The venom was treated as incompressible non-Newtonian fluid. A standard approach to describe the rheological behavior of non-Newtonian media with a shear-thinning behavior is the power-law model [16], which was applied to the current data (see below and Eq. 1) both with lower (μ_venom min_) and upper (μ_venom max_) bounds for the dynamic viscosity.
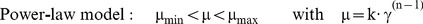
(1)Herein, 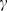 is the shear rate. The parameters of the model are adapted from the rheological measurements as follows: the minimum and the maximum dynamic viscosity bounds are 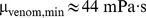 and 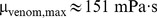, the consistency index is 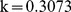 and the power-law index is 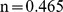.

The flow regime was assumed to be laminar because the channel geometry was of micro-scale. This is justified by estimation of the maximum Reynolds number defined for a hypothetical Newtonian case when the minimum value of dynamic viscosity μ_min_ is used and the characteristic streamwise velocity u_inlet_ at the channel inlet with an equivalent diameter of d_inlet_. The Reynolds number, which defines the ratio of inertial forces to viscous forces in the fluid [17] reads as follows:
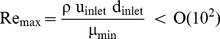
(2)with the characteristic velocity in the channel inlet given by 
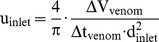
. Herein, Δt is the total venom expelling period, ΔV is the venom volume during one spit, and d_inlet_ is the inlet diameter of the channel when the cross-sectional area is calculated with a circular shape. The parameter ρ is the density of the venom. The resulting Reynolds number, Re_max_, attains values of less than 100 which is well below the critical Reynolds number of Re_crit_ = 2300, where turbulence is observed to start in channel flows [17]. Therefore, no turbulence model needs to be taken into account in the flow simulations.

A further dimensionless number to be taken into account is the so-called Strouhal number which is defined as the ratio of the characteristic time scale of the fluid, the time a fluid element needs to travel along the channel relative to the total expelling time period:
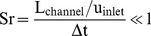
(3)where L_channel_ is the length of the venom channel. When the Strouhal number is well below unity [17] the flow process can be regarded as quasi-steady. The Strouhal number for the case of the venom channel flow has a value about Sr = 0.02. Therefore, steady-state simulations of the flow were justified. As a consequence, each phase in the flow pulse can be simulated independently. In our simulations we concentrated only on the peak flow situation where the maximum velocity is reached in the venom channel during the spitting process.

An a-priori estimation of the possible existence of secondary flow structures in the venom channel due to the strong curvature of the bend at the distal end can be discussed by means of the so-called Dean number, which has been deduced from the centrifugal flow instability in curved pipe flows [12]. The non-dimensional Dean number (Dn) characterizes the influence of these instabilities on the generation of secondary flows in a curved bend
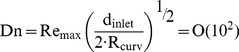
(4)where R_curv_ denotes the curvature of the bend. The Dean number, in the case of curved venom channel flow, is on the order of 100 when we use the curvature radius of the sharp bend at the distal end of the channel as a representative value (compare Table 1, see below). Because the Dean number is not low, we expect to see secondary flow structures in the results of the simulations.

**Table 1:** Parameters used for the CFD study.

Geometry		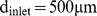
		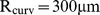
		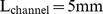
Flow parameters	Case 1 & Case 2	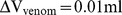
		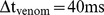

For a validation of the numerical simulations, a transparent experimental model was created. The model was scaled up 56∶1 to ease visualization of flow features. A water/glycerine mixture was chosen to match the refractive index of the transparent material of the model. The fluid exhibited Newtonian behaviour; therefore, the numerical model described in the last section was validated for the Newtonian case. In order to ensure similarity of the flow structures in the model experiment and the validation simulations, the Reynolds number was set equal. The resulting parameters of the flow in the scaled-up model are summarized in Table 2.

**Table 2:** Parameters of the scaled-up (56∶1) model case.

Geometry	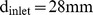
	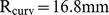
	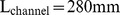
Medium	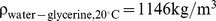
	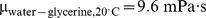
Flow velocity	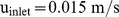

**Figure 1:**
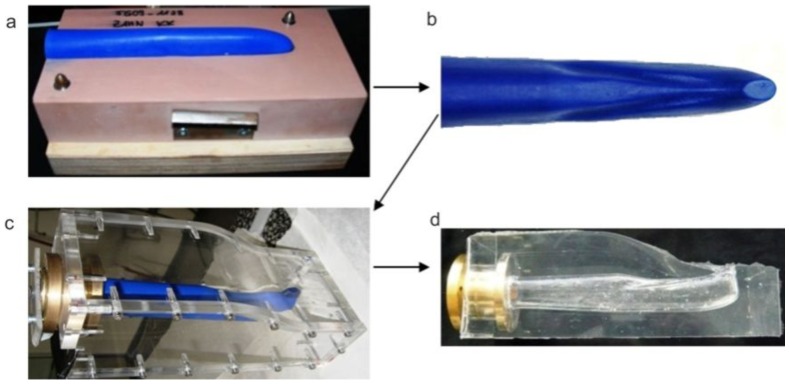
Steps for the generation of the model: mold for wax model (a), cast-wax model (b), Plexiglas casing (c), transparent-silicone model (d).

**Figure 2:**
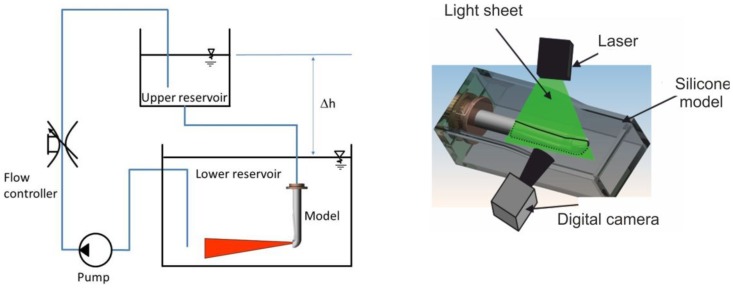
Flow circuit (left) arrangement for flow measurements inside the model of the fang (right).

The steps for the generation of the transparent model are given in Fig. 1. With the aid of the MCT-data, a negative form was created out of wax. In a further step, silicon was casted around the wax form and the wax then removed, resulting in a transparent model of the cobra's venom channel. The experimental measurements were carried out in the test facility described in Fig. 2 and below. The facility consisted of an upper and a lower reservoir, which were interconnected on one side by the feed flow and on the other side by the return flow. The silicone model was integrated into the lower reservoir and supplied by the liquid from the upper reservoir. To assure constant inflow and outflow conditions, the height difference Δh between the two reservoirs was kept constant. A light sheet (about 2 mm thick) was generated by a laser (New Wave, Pegasus, high-speed, dual cavity, 10 mJ @1 kHz) and light sheet optics. The particles (Vestosint, Evonik Degussa GmbH, mean diameter 20 µm) in the flow were illuminated by the laser sheet and filmed with a high-speed camera (Photron, APX RS , 1024×1024pix^2^ @ max 3000 fps), which was arranged perpendicular to the light sheet. An area of about 50×50 mm was captured with a separation time of 200 µs at 1000 Hz. The post-processing of all numerical and experimental results was carried out in TECPLOT 360 (Tecplot Inc.).

## Results

### Physical-chemical properties of the venom

**Figure 3:**
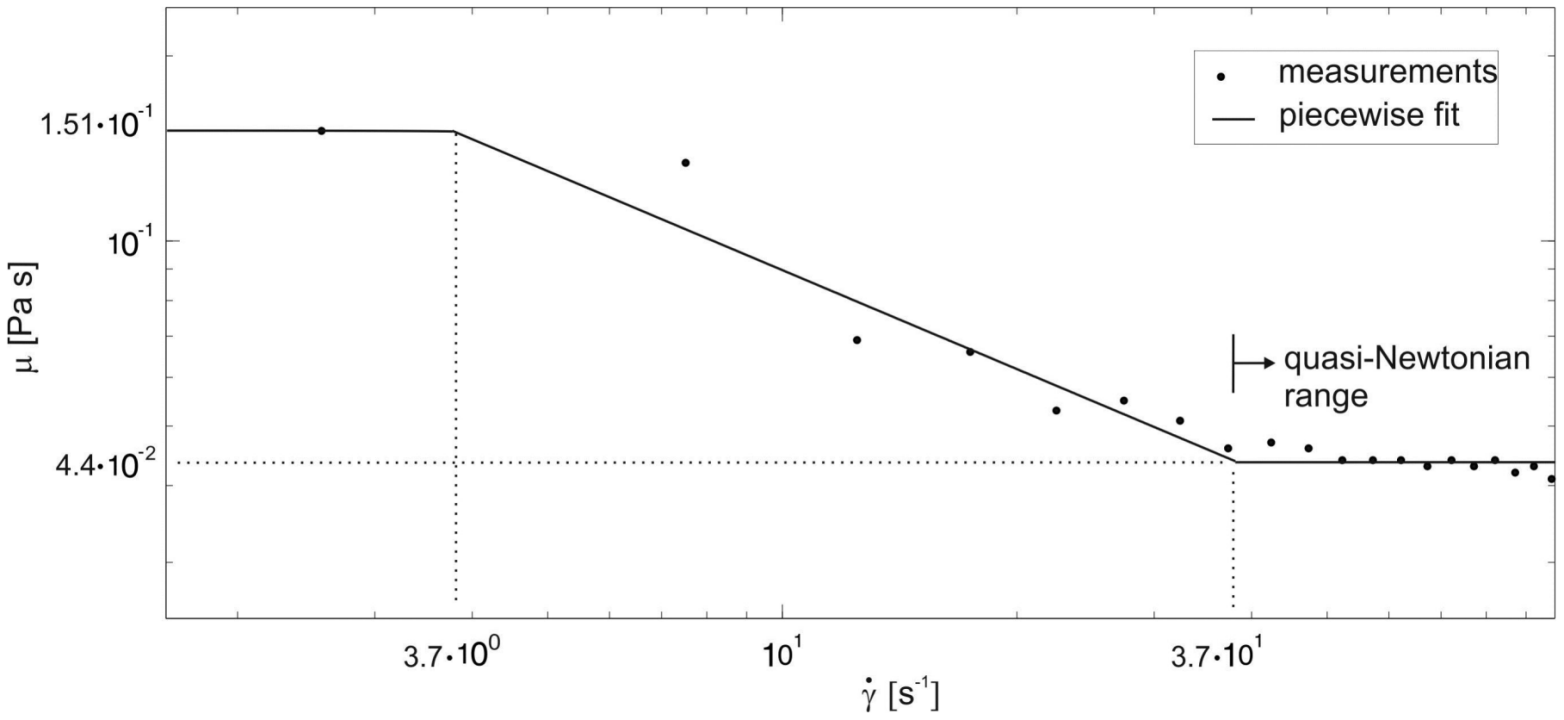
Average viscosities of the venom of *N. pallida* (dots) as function of shear rate 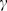 (s^−1^) expressed on a logarithmic scale. Note that the viscosity decreased with increasing shear rate and remained nearly constant for shear rates >3.7 10^1^ s^−1^.

For the determination of the physical-chemical properties of the venom, each cobra (N = 7) was milked two to three times. The venom volume obtained in a single milking process was 0.46 ml±0.13 ml. At low shear rates, viscosity was high, usually between 0.1 and 1.1 Pa s. For shear rates lower than about 37 s^−1^, a shear thinning behavior was observed (Fig. 3). For higher shear rates, the viscosity did not change and remained in a quasi-Newtonian range. The minimum and the maximum dynamic viscosity bounds are 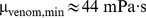 and 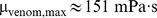. Viscosity values were comparable when successive measurements were performed on the same sample (the time interval between measurements was five minutes).The surface tension of the cobra venom was lower (60 mN/m±5 mN/m) than the surface tension of water (20°C, 70 mN/m). The density of the venom was 1084 kg/m^3^±25 kg/m^3^. This value is close to the density of water (1000 kg/m^3^), whereas the viscosity in the Newtonian range was 44 times higher than the viscosity of water.

### Spitting volume and spitting period

These data were taken from earlier studies in our group and were cross-checked with new experiments. A single spit of *N. pallida* contains at least 2% of the venom gland volume (see also below, [18], [15], [19]). At each milking process, we obtained an average volume of 0.5 ml (see results), which we assumed to be the average venom gland volume. A single spit can therefore have a volume of at least 0.01 ml up to about 0.5 ml that is expelled in 40/70 ms [11], [15]. However, our assumption was that a spitting cobra will not eject all its venom in one spitting act. Therefore, we estimated the maximum volume of a venom jet to be 0.1 ml. An average of 40 ms was taken as a reference value for the spitting time.

### Morphology of the venom channel

**Figure 4:**
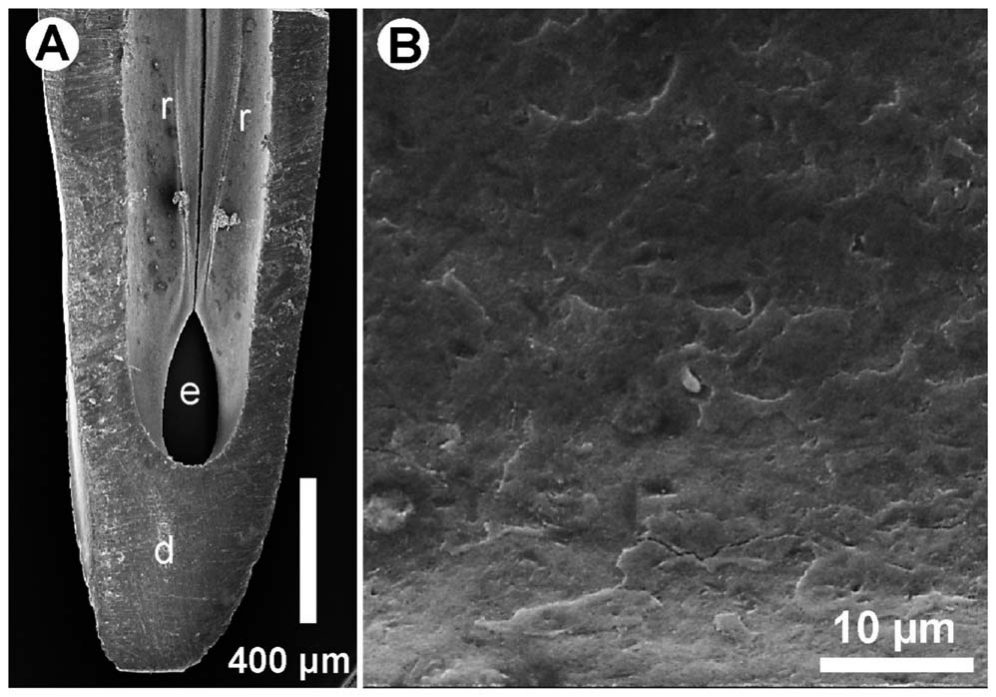
SEM image of a parasagittal section (d = dentin) of a *N. pallida* fang. (A) The exit orifice (e) as well as the symmetrical ridges (r) are displayed. (B) SEM image of the inner surface of the channel in high magnification. The surface is smooth in the micron dimension. No microstructures are visible that might affect the venom flow.

**Figure 5:**
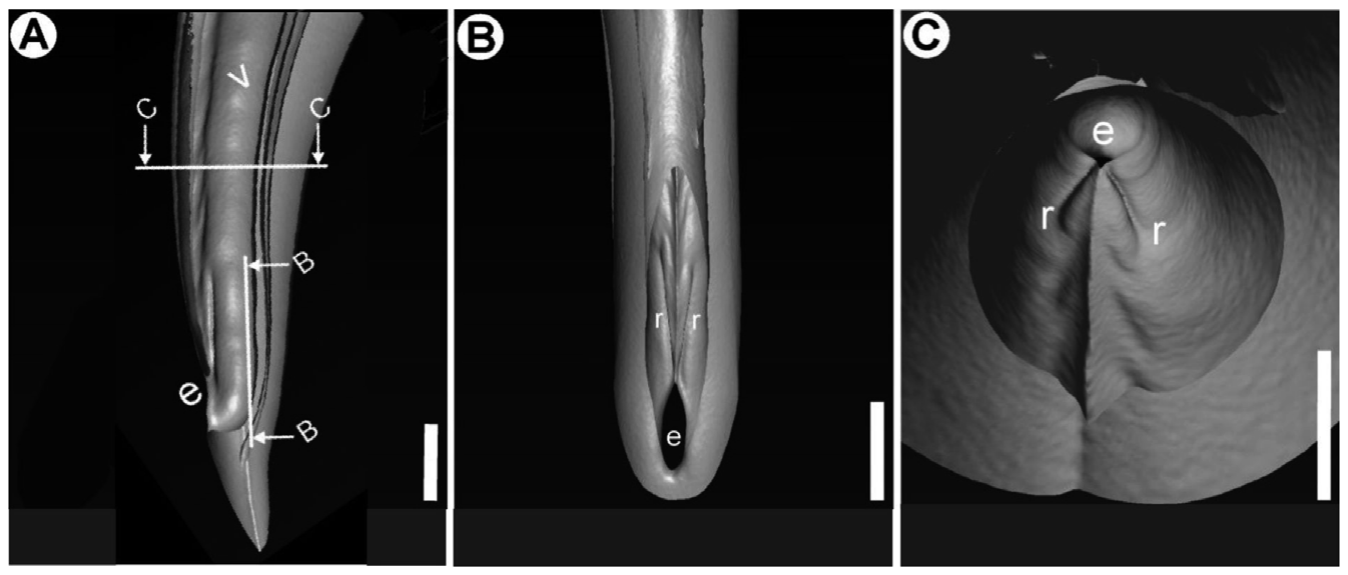
3d-reconstruction of a *N. pallida* fang obtained from MCT data. (A) Lateral view into the venom channel (v). The cutting planes and viewing angles of images B and C are indicated. (B) Dorsal view towards the exit orifice (e). Note the symmetrical ridges (r) above the exit orifice. (C) View from the middle of the venom channel towards the exit orifice, which is oriented downwards in this view. Scale bars: 500 µm (A, B) and 200 µm (C).

The MCT scans delivered a sufficiently high resolution of the venom channel of the cobra fangs. Average voxel size was 7 µm^3^ with high contrast. All characteristic structures, like the prominent ridges that were visible in the SEM (Fig. 4), were also visible in the MCT Scans (cf. Fig. 5). The venom channel of *N. pallida* featured two internal ridges on the ventral surface and a sharp and tapered turn close to its exit. The ridges were symmetrical and protruded up to 50 µm into the channel lumen. They covered about 1/5 of the channel length. Besides the ridges, the surface of the venom channel was rather smooth and did not contain any microstructures in the SEM (Fig. 4). The 3D structure of the venom channel of a cobra fang was reconstructed from the images of cross-sections and then smoothed mathematically. This geometry of the venom channel is then used for the flow studies as internal channel configuration.

### CFD simulations

**Figure 6:**
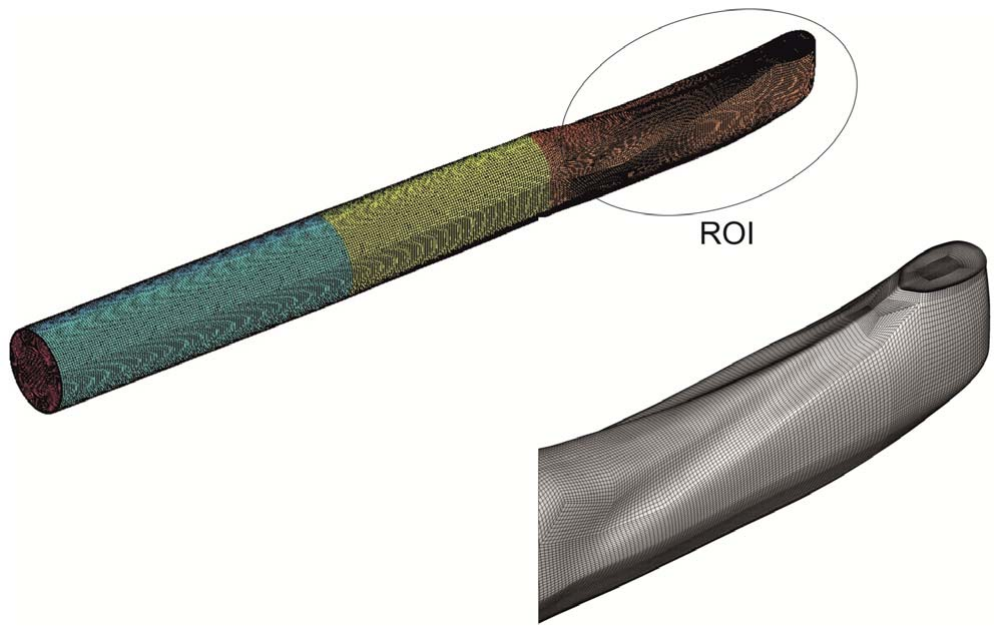
Computational domain and mesh topology in the region of interest (ROI) of the venom channel.

The numerical flow simulation using CFD was performed with the commercial software FLUENT (ANSYS Inc., see Appendix S1). Boundary conditions were applied in agreement with the experimental investigations and the original case (see Table 3). For a prescribed flow, we obtain the pressure difference 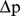 between inlet and outlet boundary. In the CFD simulations the outlet pressure is set to zero as a reference value. For the CFD simulations, fluid properties and the mean flow velocities were obtained from the spitting tests (see Table 1). The computational domain, as marked in Fig. 6, includes three segments: a circular inflow tube, a transitional region which fits smoothly the tube with the inlet of the venom channel and finally the venom channel with the region of interest consisting of the two ridges and a tapered bend at the exit to the ambient. This geometry is referenced in the following as Case 1. Another geometry of the venom channel was generated as a reference case (Case 2) where the ridges are removed while keeping all other inner geometries and scales the same as in Case 1. This allows us to compare the flow structures in the venom channel with ridges against the same venom flow channel but without ridges.

**Table 3:** Boundary conditions used for the simulations.

boundary	Variable	Type	condition
inlet	*velocity*	*Dirichlet*	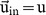
	*pressure*	*Neumann*	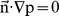
outlet	*velocity*	*Neumann*	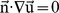
	*pressure*	*Dirichlet*	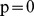
wall	*velocity*	*Dirichlet*	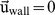
	*pressure*	*Neumann*	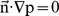

**Figure 7:**
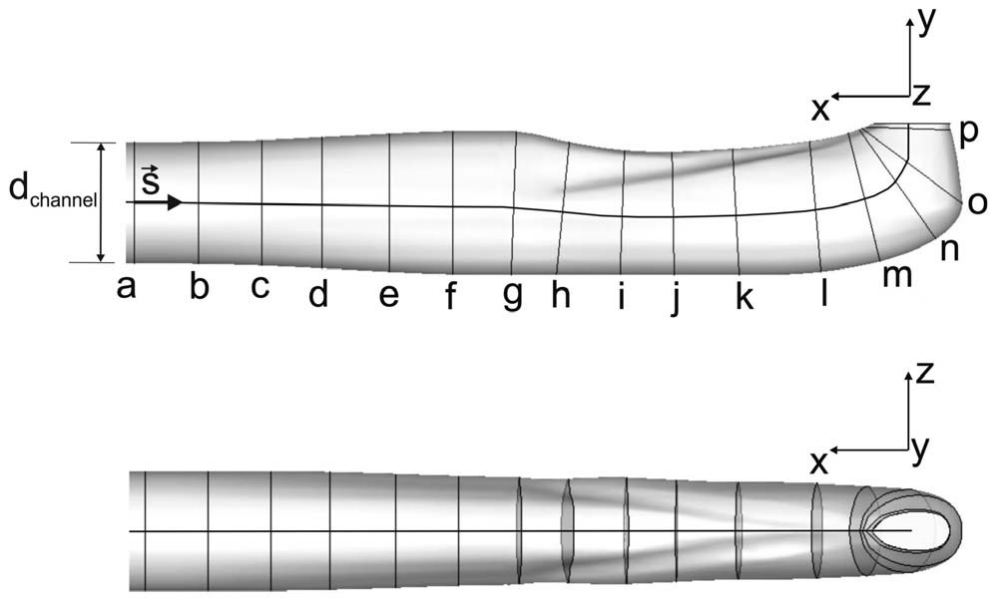
Cross-section positions at various downstream locations: lateral (top) and top view (bottom). S indicates the midline of the channel. Note the curvature of the midline in the top graph.

Due to its complex geometry, the channel (cf. Fig. 7) was subdivided into three parts:

Part 1 (a–f): comprising transition from circular to quasi-elliptic cross-section.

Part 2 (g–k): where the influence of the curvature and the two ridges on the flow is expected.

Part 3 (l–p): including a sharp turn bend with a tapered cross-section.

**Figure 8:**
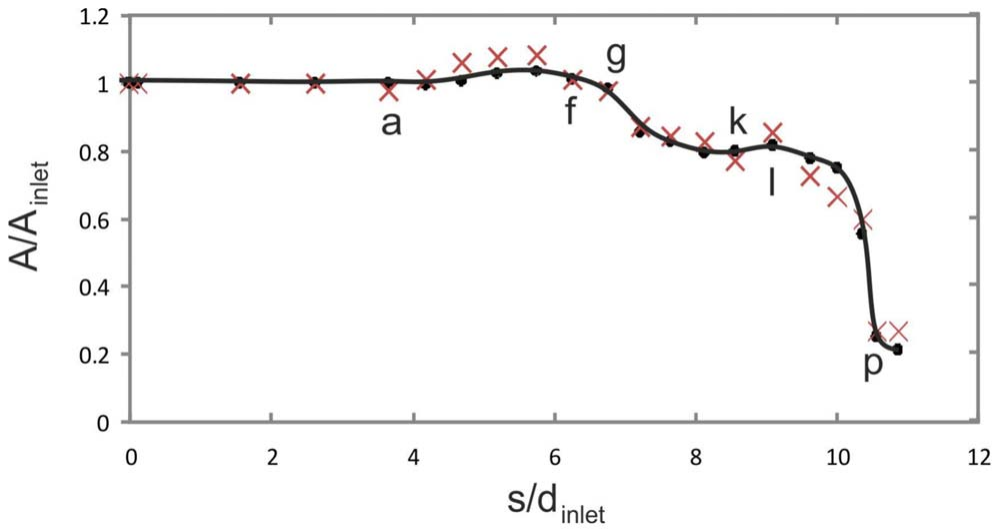
Normalized cross sectional area along the midline (s) of the channel. Solid line/dots: venom channel with ridges; crosses: venom channel without ridges (reference case); A_inlet_ = 1.963e-07 m^2^; a to p: Positions of cross sections are indicated in Fig. 7.

Fig. 8 shows the changes in the cross-sectional area A of the venom channel. The values are made dimensionless by diving A by A_inlet_ of the cross-section at the inlet. Dots correspond to the discretized locations along the midline of the channel shown in Fig. 7. The graph shows two taperings within the channel: the first tapering corresponds to the drop in the cross sectional area of about 20% in the region between (g) to (i); the second tapering to the drop in the cross sectional area of about 60% in the region between (l) to (p). Crosses correspond to a venom channel that lacks ridges but has an elliptical cross-section of almost equivalent areas (reference case).

**Figure 9:**
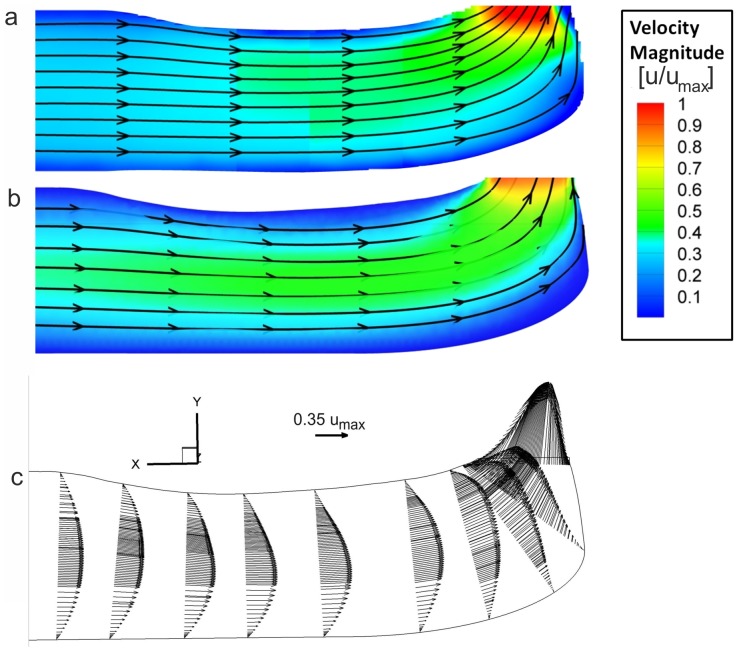
PIV measurements of the flow (A) and CFD results of the venom flow (B). Graphs show sectional streamlines in the mid-coronal cross-section and the contour of the velocity magnitude. (C) CFD result: vectors indicate the streamwise velocity profiles. A transformation of the velocity profile along the venom channel is visible.

Fig. 9A shows the velocity field in the frontal midplane of the fang model obtained in the experiments, and Fig. 9B displays the results from the numerical simulation. Note that the maximum fluid velocity occurred close to the exit orifice plane (cf. Table 4). Similar velocity distributions and sectional streamline patterns were apparent in both cases. The streamlines indicate the jet (expelling) angle distribution along the exit area. The reshaping of the axial velocity profiles can be seen. The typical parabolic profile for pipe flow changed along the venom channel and was influenced by the geometry of the channel.

**Table 4:** Calculated pressure and velocity values for Cases 1 and 2.

	Case 1	Case 2 (reference)
Δp_inlet-outlet_ (10^6^ Pa)	*0.1140*	*0.0960*
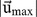 (m/s)	*9.27*	*7.43*
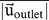 (m/s)	*6.32*	*4.98*
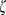	*4.307*	*6.207*

The values for the pressure build-up and the maximum liquid velocities are summarized in Table 4. From conservation of mass, we expected higher velocities at the exit orifice relative to the values at the inlet due to the decrease of cross-sectional area. As a consequence, pressure of an ideal fluid (zero viscosity, no energy loss) must decrease because of the increase in velocity. However, in real fluids an additional pressure drop is present due to wall friction, generation of secondary flows, and flow separation (energy loss). In order to compare the resulting energy loss for the different cases of the flow channel, we introduced a loss coefficient ζ defined as:
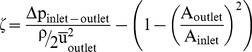
(5)This coefficient allows us to compare the energy loss in the different flow channels, because it excludes any influence of the static pressure differences at the orifice due to slightly different cross-sectional areas A_outlet_ in Cases 1 and 2. As seen from the results, the loss coefficient is about 30% lower in a venom channel with ridges than in the venom channel without ridges (reference case), which is a major result of our study.

**Figure 10:**
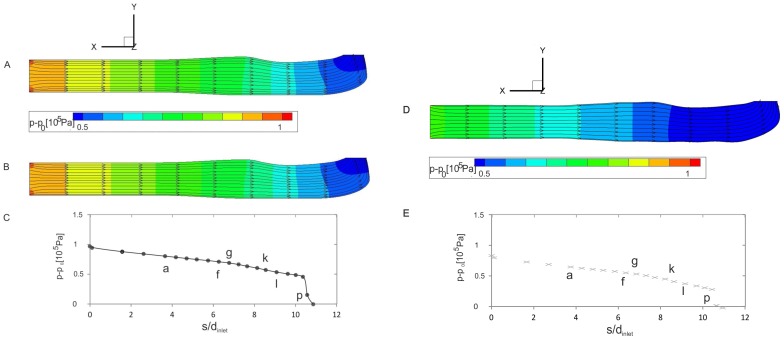
Flow along the venom channel. Case 1: pressure distribution and streamlines in the frontal midplane for the Newtonian (A) and the non-Newtonian case (B). (C) Integral pressure in the sections along the midline s of the channel for the Newtonian case (cf. Fig. 7). Reference case: pressure distribution and streamlines in the frontal midplane for the Newtonian (Case 2 = reference) case (D). (E) Integral pressure in sections along the midline s of the venom channel (cf. Fig. 7).

In Fig. 10, the Newtonian case is compared with the non-Newtonian simulation case. No significant differences exist between the two flow regimes. The change of the pressure distribution along the channel in the midplane is shown in Figs. 10C and 10E.

**Figure 11:**
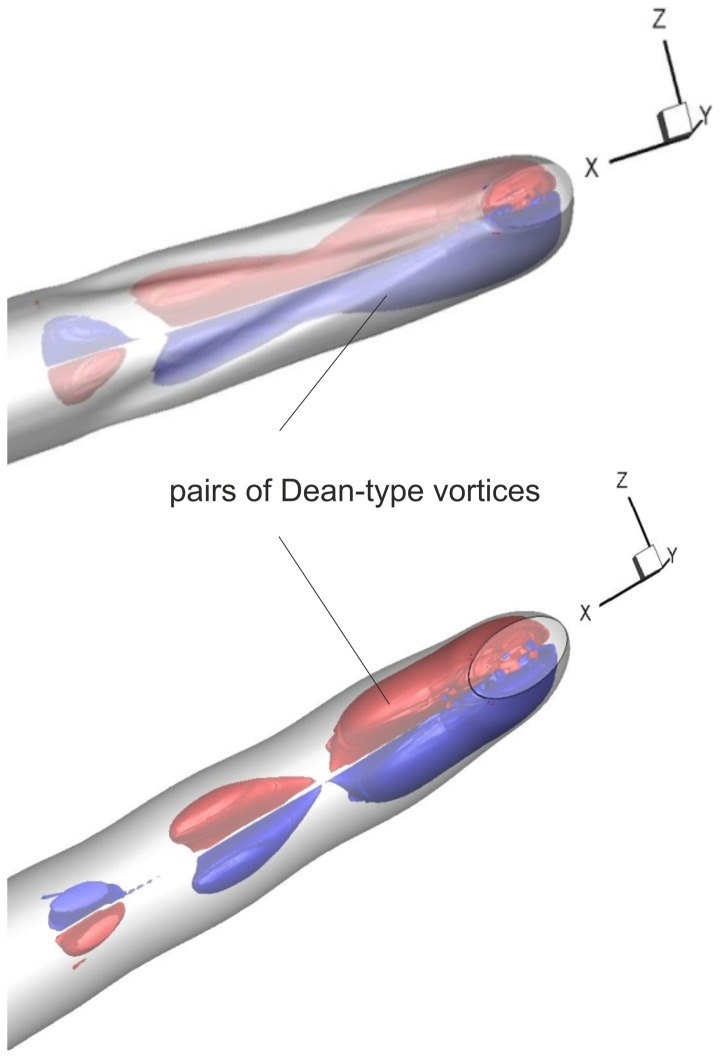
Isosurface plots of regions of concentrated helicity indicating the pre-conditioning and generation of secondary flow structures for Case 1 (top), and 2 (bottom). The normalized helicity is +0.05 (red) and −0.05 (blue).

In order to visualize the secondary flow structures, we used the value of helicity. Levy et al. [20] introduced the term helicity, which is used for the detection of streamwise-oriented vortex cores. In normalized form, helicity is defined as:
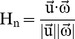
(6)with **u** = velocity and **ω** = vorticity vector. Near the vortex center, the angle between these two vectors is small in the case of streamwise-oriented vortices such as the Dean-type vortices, thus the helicity is high. The normalized helicity has limiting values of ±1, where the angles between the velocity and vorticity are zero, and the sign depends on the direction of rotation. To get a 3D picture of the vortical structures within the channel, two surfaces of constant normalized helicity are shown in Fig. 11. A change in the direction of rotation is seen in the region of the ridges.

**Figure 12:**
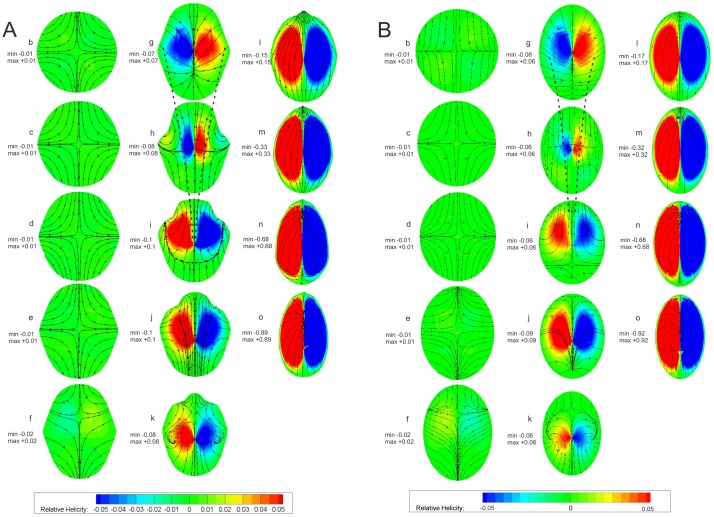
Cross-sectional distribution of helicity with sectional streamlines at various downstream locations (cf. Fig. 7): blue −0.05, red +0.05. Min. and maximum values of relative helicity are given. Case 1 (A) and reference case (B).

Figs. 12A and 12B show the flow in the sections defined in Fig. 7. The sectional streamlines are plotted with the normalized helicity that is color-coded. The channel was divided according to its geometry and the appearing flow structures into the segments a–f, g–k and l–o. The development of secondary flow was reflected in flow topology, such as saddle points, spirals, and centers. At the end of segment a–f, which marks the transition of the circular into a quasi-elliptical cross-section of the same cross-sectional area, a saddle point was evident in the center of the channel. It was detected due to a widening in the vertical direction and the resulting slight diffuser effect. Already in section f, the upstream effect of the curvature and the two ridges was present. This is more pronounced in the segment g–k.

### The functional significance of the venom channel ridges

The comparison of Case 1 (venom channel with ridges) with Case 2 (venom channel without ridges) evidences the effect of the ridges. The ridges (Case 1) cause a smooth and gradual increase of the helicity in magnitude and extent during the generation of the Dean type vortices of the sharp bend (cf. Fig. 12A and 12B, h–k). Section g shows the emergence of two counter-rotating vortices just before the ridges are formed. This was due to the slight clockwise curvature of the channel in this section. From g to h the cross-sectional area was reduced by 20%. This caused a nozzle effect, seen in the path direction of the sectional streamlines. The helicity remained at a low level. The borders of the preconditioning structures generated before the ridges are indicated by the dashed lines. In the segment l–p the level of secondary flow in Case 1 remained low until reaching the exit orifice. Secondary flow was generated due to the centrifugal force in the bend (Dean vortices). These vortices had a sense of rotation equal to the one generated by the ridges. A strong velocity component perpendicular to the streamlines emerged, which was indicating stronger streamwise vortices: in the core, this secondary flow was directed to the outer wall of the bend while along the walls it was directed towards the inner part of the bend. At the outlet, vortices were no longer visible. The high helicity values were mainly caused by the high axial velocity rather than the small radial and tangential velocity components. Compared to the reference case, in Case 1 the gradual decrease of the counter-rotating vortex structures and the gradual increase of the subsequent Dean-type vortices was more pronounced. This suggested that the ridges are relevant to the formation of a secondary flow structure upstream of the sharp exit bend in the same sense of rotation as the Dean-type vortices would be formed farther downstream in the bend. Therefore, the ridges effectively enlarge the curvature radius because secondary flow is already formed upstream of the bend.

**Figure 13:**
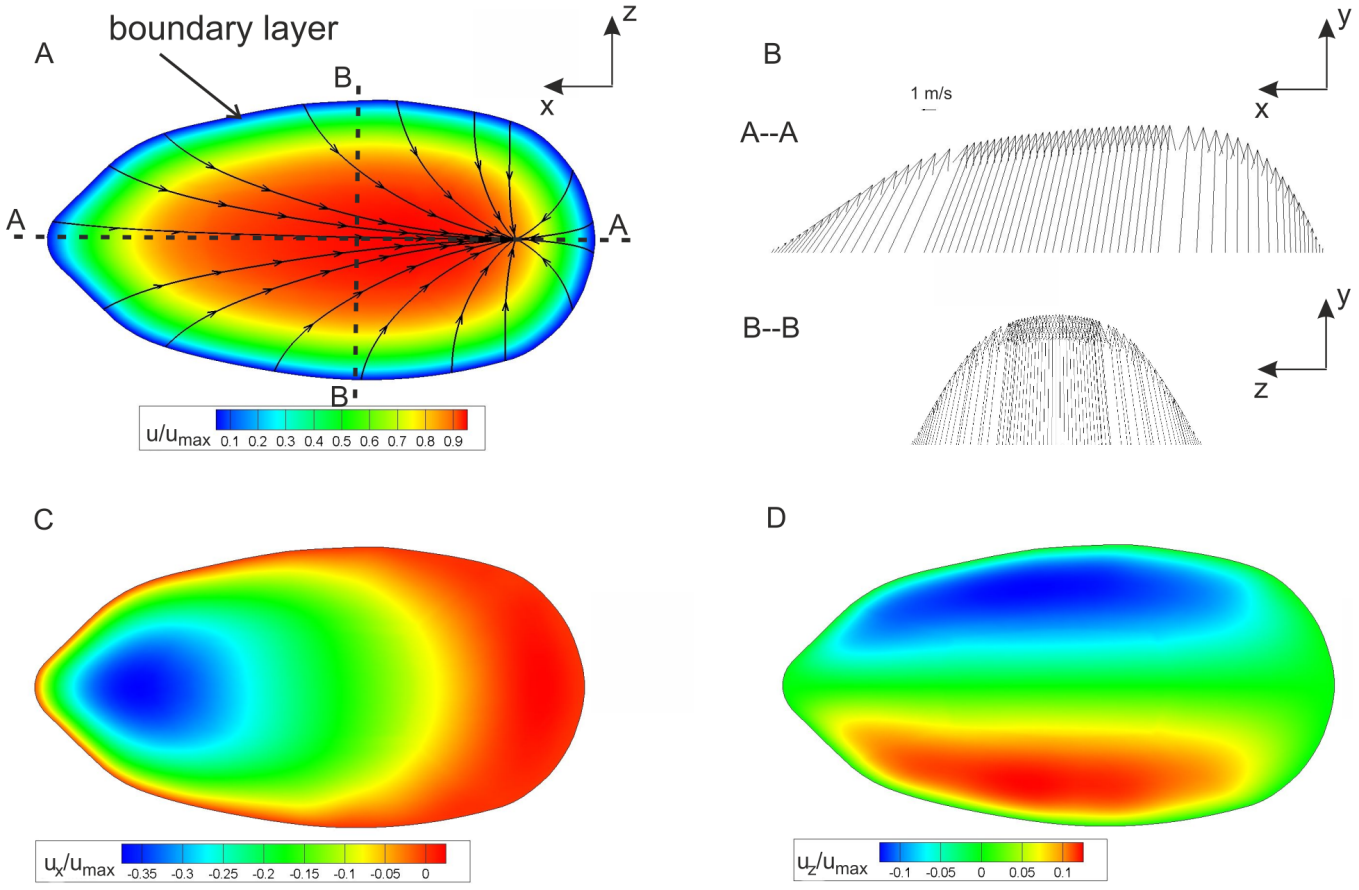
Case 1: (A) Flow structures at the outlet. The velocity magnitude is color-coded. (B) Velocity profile plots in sections A and B from (A). Contours of velocity components (color-coded) u_x_ (C) and u_z_ (D) at the outlet plane.

**Figure 14:**
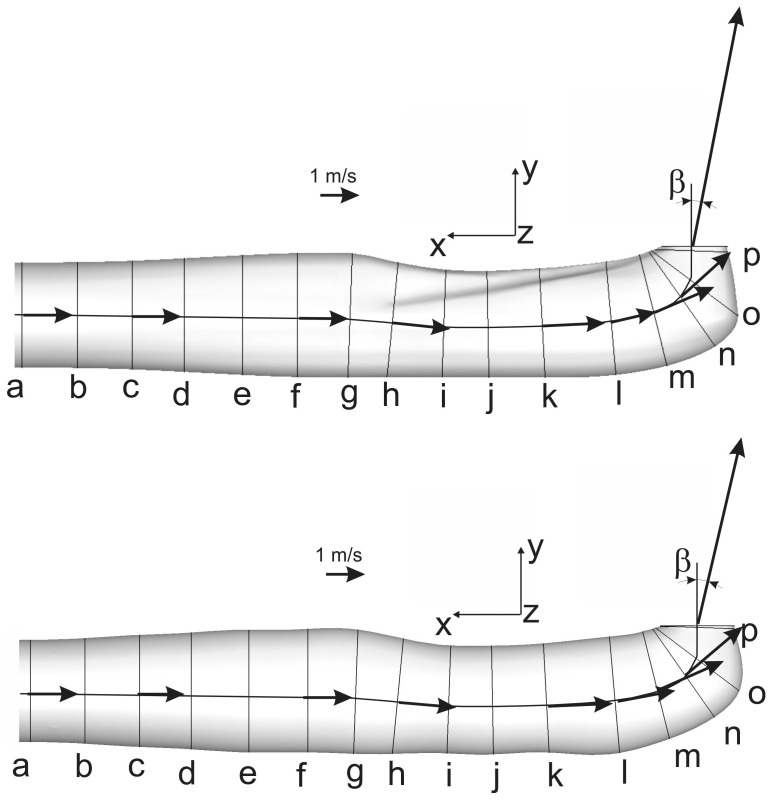
Mean velocity (arrow length) of the liquid in the sections given in Fig. 7 for Case 1 (top) and reference case (bottom).

Another indication of the strength of the secondary vortices and their influence on the flow at the orifice is the so-called slip. This is the deviation of flow direction from the channel axis at the exit. The flow at the exit orifice is mapped in Fig. 13. The velocity profiles in two distinct sections are shown. The slip of the flow is primarily caused by the secondary flow in the bend. A higher slip is synonymous with stronger secondary flows. In order to determine the slip quantitatively, we calculated the mean deviation angle of the velocity vectors β at the outlet cross-section (orifice plane) relative to the center-axis of the orifice plane. The results showed a deviation angle of 11.1° for Case 1 and 13.2° for the reference case (cf. Fig. 14). Hence, the reference case showed a stronger action of secondary flows disturbing the exit flow direction than Case 1 including the ridges. This provides additional confirmation for the higher pressure loss in a channel without ridges and thereby identifies the ridges as the potential source of the reduction in pressure loss.

## Discussion

The focus of the paper was the analysis of the venom flow in the venom channel of a spitting cobra using PIV and CFD in a model of the channel. First, we measured the rheological behavior of the venom fluid. The results confirm trends documented by Balmert et al. [21] and Young et al. [22], i.e., that the venom showed a shear-thinning behavior. This implies that flow is less viscous at high shear rates. The shear-thinning property of the venom may be important for cobras, or even for all snake species with closed-grooved fangs: the venom stored in the venom glands and the fangs has a high viscosity, and therefore flows only slowly if no pressure is applied. This prevents the venom from unintentionally leaking out of the fangs. While spitting, the *musculus adductor mandibulae externus superficialis* contracts, thus increasing the pressure in the venom gland [15]. The venom is then pushed through the venom channel and the shear rate rises. Thus, the viscosity of the venom decreases and therefore the venom can be discharged more easily and at higher velocities.

The possible relevance of this non-Newtonian behavior of the cobra venom was studied using CFD with a prescribed power-law of the liquid viscosity approximating the measured rheological properties of the venom. When comparing the CFD results for non-Newtonian and Newtonian behavior, we could detect only marginal differences in flow structure. Therefore, we conclude that for the major time-span of the spitting process, when fluid is already set into motion, non-Newtonian effects do not have any grave influence on the flow structures. Flow structures for Newtonian case are in good qualitative and quantitative agreement when comparing CFD and PIV results.

The internal flow in the venom channel is characterized by the generation of secondary flow structures caused by the combination of the ridges and the bend located farther downstream. The channel terminates into the orifice at an angle of roughly 90° relative to the main axis of the fang. Thus, the bend is rather sharp. Ridges are positioned at the channel wall in line with the plane of the nozzle exit and farther upstream from the bend. In addition, the channel demonstrates a high degree of planar symmetry to the center plane. The reconstruction of the helicity distribution in the channel shows that the small S-type curvature in a clockwise direction generates a secondary flow structure in form of two counter-rotating streamwise vortices. The sense of rotation of the vortex pair is opposite to the Dean-type vortices generated farther downstream in the sharp bend. Thus, the flow upstream of the bend is pre-conditioned such that it reduces the effect of the sharp bend on the flow at the nozzle exit. This is reminiscent of a mechanism known from serpentine channels in which the direction of the curvature is successively reversed to reduce the effect of secondary flow [23]. A further effect is achieved by the ridges, which form the entrance to the bend. According to our results, the ridges generate a pair of streamwise vortices in the same sense of rotation as the - further downstream in the bend - generated Dean-type vortices. This vortex pair is named in the following the pre-cursor vortex pair to distinguish it from the Dean-type vortex pair. The strength of the vortices seems to be somewhat smaller than those of the Dean-type vortices. As the quantitative results of the pressure drop along the venom channel show, the presence of the pre-cursor vortices due to the action of the ridges decreases the pressure drop. This is also proven by the reduced slip at the exit of the venom channel. Therefore, we claim that the ridges act similar to the function of guide vanes that are used by engineers to reduce pressure loss in a curved bend. As the MCT geometries of the venom channel let recognize (Fig. 5), some of the microscan contours of the cobra fang demonstrate the successive appearance of paired ridges in the channel in 2–3 successions. This indicates that the strength of the pre-cursor vortices could be adapted via the number of ridges and their protrusion into the channel.

The main results can be summarized as follows: No significant effect of the non-Newtonian fluid behavior was seen inside the channel when comparing to the Newtonian case once the flow was established in the channel. The importance of geometry (ridges, sharp tapered bend) has been shown. The two ridges, forming the entrance into the sharp turn, generated - as a precursor - a secondary motion that interacted with the curved flow in the sharp bend in a beneficial way such that pressure loss was reduced by about 30% compared to an identical channel without ridges. The higher mean flow velocity at the outlet orifice helps the cobra to achieve a longer reach of the jet. The ridges therefore play an important role, which could be compared to the method of engineers to reduce pressure loss in curved flows by implementing guide vanes. It is shown from the first developments of Göttingen-type wind tunnels that such vanes in the bends reduce the effect of secondary flows and pressure loss [17].

## Supporting Information

Appendix S1 **Mesh information and equations of conservation of mass and momentum.**
(DOCX)
