# Supplementary material for: 3D Flow in the Venom Channel of a Spitting Cobra: Do the Ridges in the Fangs Act as Fluid Guide Vanes?
Source: PLoS One. 2013 May 6;8(5):e61548. doi: 10.1371/journal.pone.0061548 (PMC3645995; doi:10.1371/journal.pone.0061548)
Supplement: Appendix S1 — Mesh information and equations of conservation of mass and momentum. (DOCX) [file pone.0061548.s001.docx]

A block-structured mesh of 1.5 million cells was implemented. The angles of the joints were all above 30° and the value of the determinant of the Jacobian matrix was higher than 0.5, following convergence considerations [1]. This is standard procedure in CFD. The code FLUENT (ANSYS Inc.) numerically solves the equations of conservation of mass and momentum (Eqs. (S1)) by using a finite-volume approach.

**
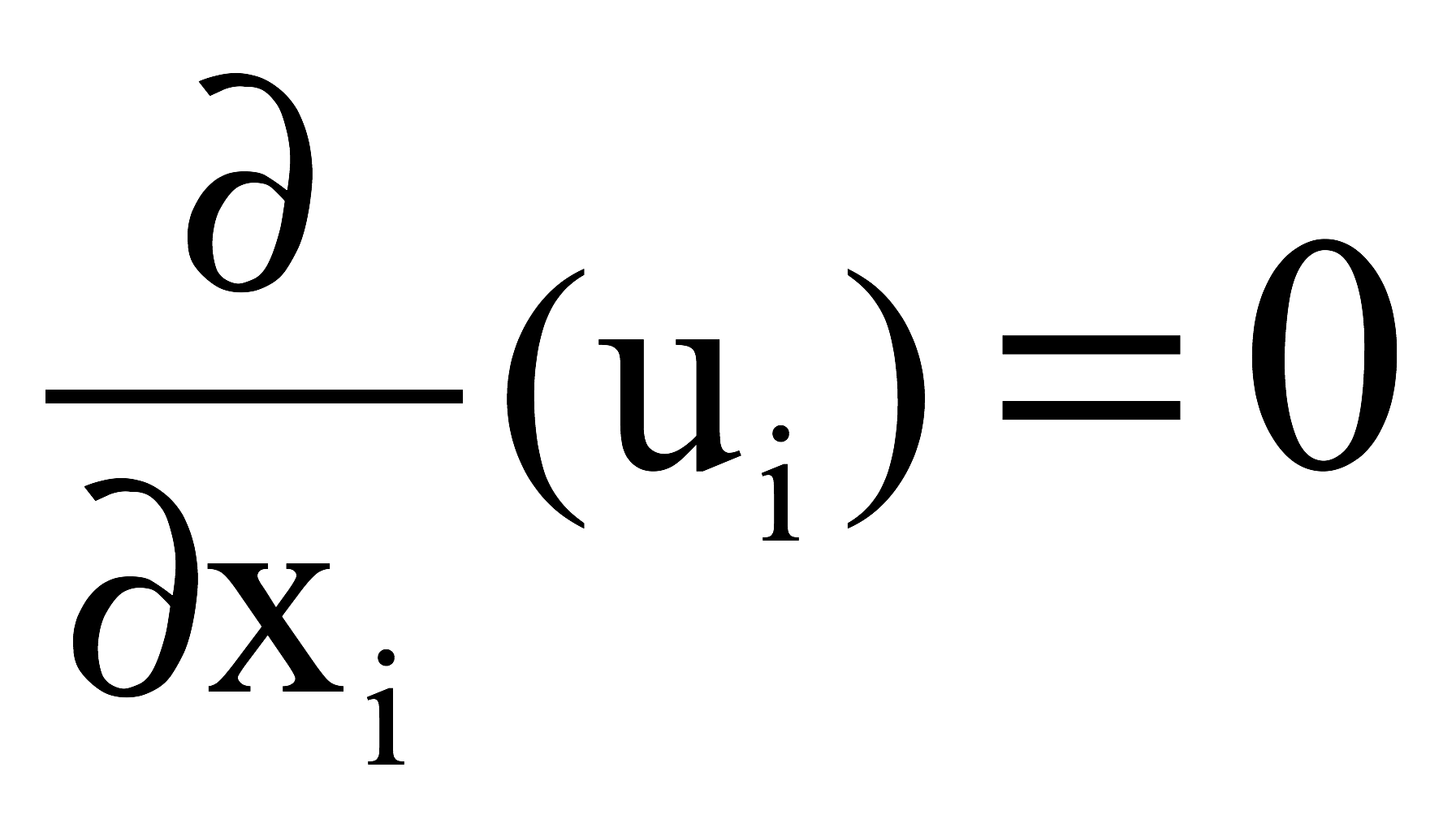
** , **
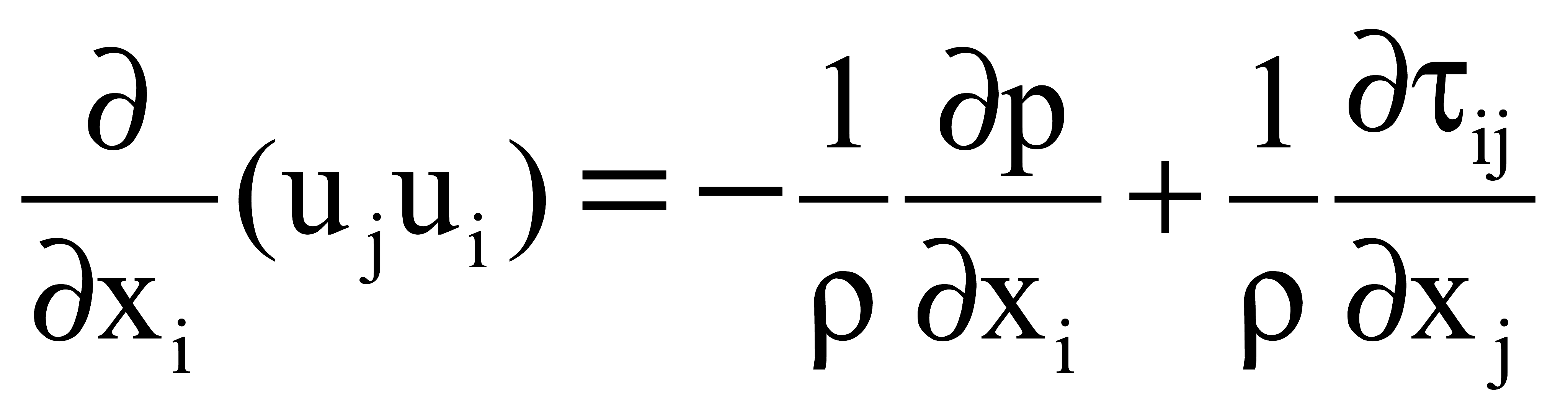
** (S1)

The shear stress τ_ij_ can be expressed in terms of the non-Newtonian viscosity μ and the rate-of-deformation tensor s_ij_:

**
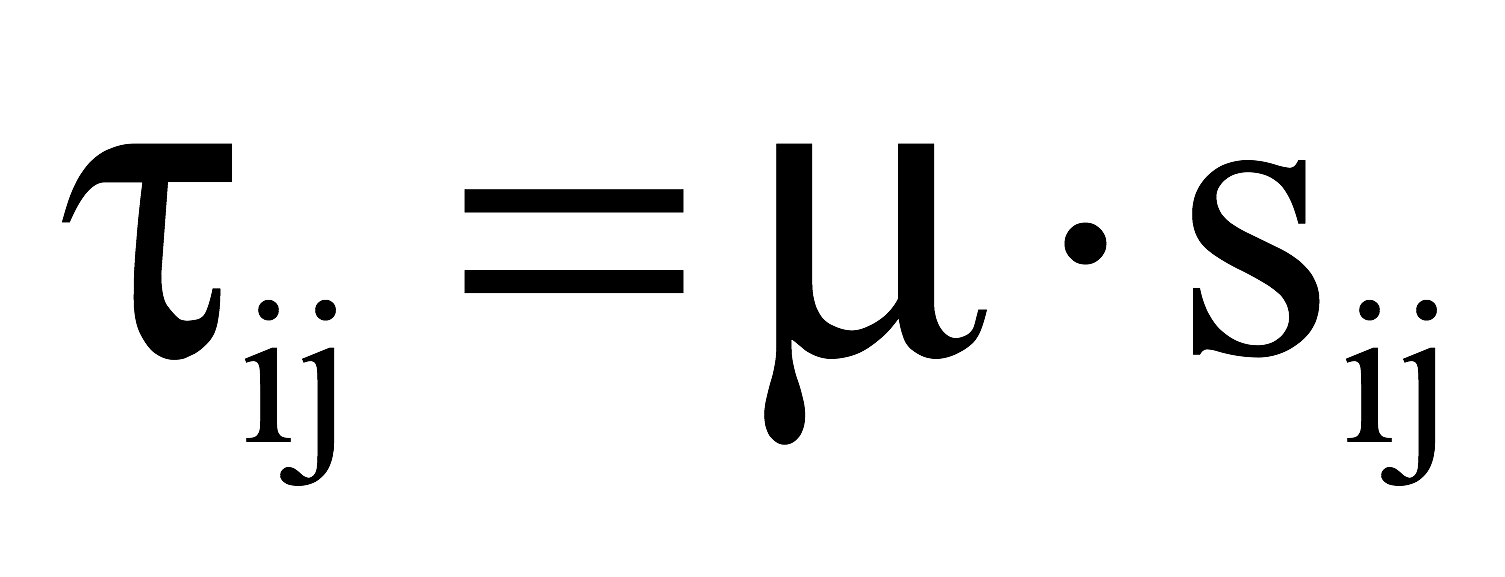
** (S2)

**
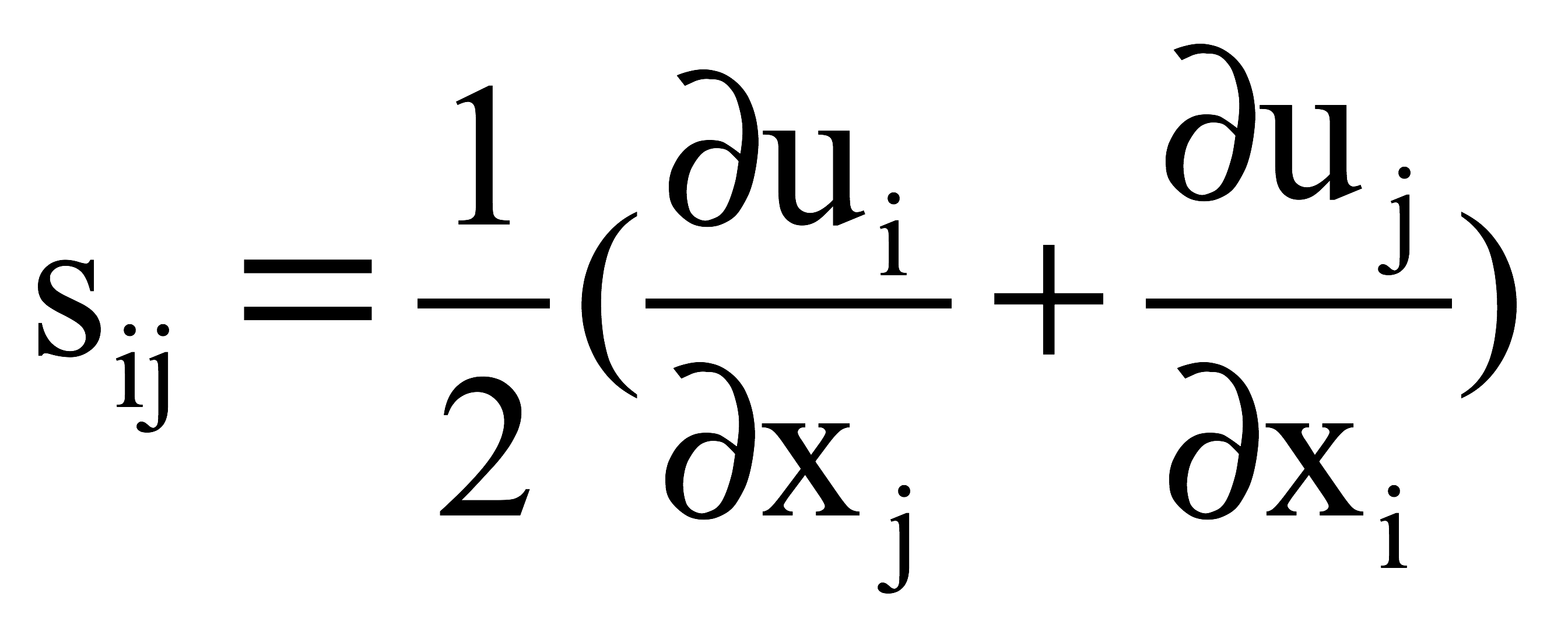
** (S3)

and μ from Eq. 1 with

**
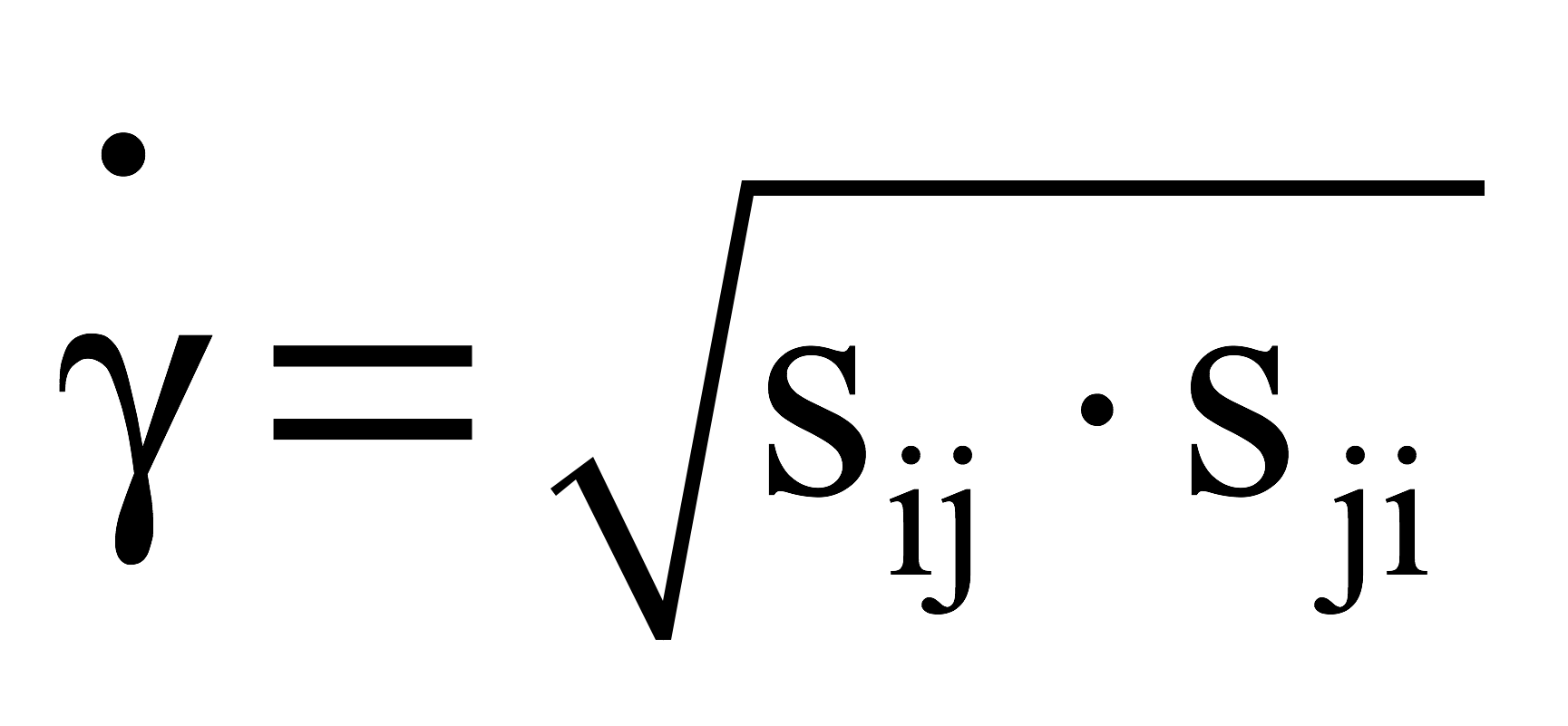
** (S4)

**Supporting References**

1. ANSYS 12.1 ICEM CFD (2009) User manual
